# Mass Drug Administration With High-Dose Ivermectin and Dihydroartemisinin-Piperaquine for Malaria Elimination in an Area of Low Transmission With High Coverage of Malaria Control Interventions: Protocol for the MASSIV Cluster Randomized Clinical Trial

**DOI:** 10.2196/20904

**Published:** 2020-11-19

**Authors:** Edgard Diniba Dabira, Harouna M Soumare, Steven W Lindsay, Bakary Conteh, Fatima Ceesay, John Bradley, Christian Kositz, Henk Broekhuizen, Balla Kandeh, Alexandra E Fehr, Claudia Nieto-Sanchez, Joan Muela Ribera, Koen Peeters Grietens, Menno Roderick Smit, Chris Drakeley, Teun Bousema, Jane Achan, Umberto D’Alessandro

**Affiliations:** 1 Medical Research Council Unit Gambia London School of Hygiene and Tropical Medicine Banjul Gambia; 2 Department of Biosciences Durham University Durham United Kingdom; 3 London School of Hygiene and Tropical Medicine London United Kingdom; 4 Radboud University Medical Centre Nijmegen Netherlands; 5 National Malaria Control Program, The Gambia Banjul Gambia; 6 Medical Anthropology Unit Department of Public Health Institute of Tropical Medicine Antwerp Belgium; 7 Vrije Universiteit Amsterdam Athena Institute Amsterdam Netherlands; 8 Medial Anthropology Research Center Universitat Rovira i Virgili Tarragona Spain; 9 Amsterdam Centre for Global Child Health, Emma Children’s Hospital, Amsterdam University Medical Centres, Amsterdam, The Netherlands Amsterdam Netherlands; 10 Liverpool School of Tropical Medicine Liverpool United Kingdom

**Keywords:** ivermectin, dihydroartemisinin-piperaquine, mass drug administration, malaria, cluster randomized trial, The Gambia

## Abstract

**Background:**

With a decline in malaria burden, innovative interventions and tools are required to reduce malaria transmission further. Mass drug administration (MDA) of artemisinin-based combination therapy (ACT) has been identified as a potential tool to further reduce malaria transmission, where coverage of vector control interventions is already high. However, the impact is limited in time. Combining an ACT with an endectocide treatment that is able to reduce vector survival, such as ivermectin (IVM), could increase the impact of MDA and offer a new tool to reduce malaria transmission.

**Objective:**

The study objective is to evaluate the impact of MDA with IVM plus dihydroartemisinin-piperaquine (DP) on malaria transmission in an area with high coverage of malaria control interventions.

**Methods:**

The study is a cluster randomized trial in the Upper River Region of The Gambia and included 32 villages (16 control and 16 intervention). A buffer zone of ~2 km was created around all intervention clusters. MDA with IVM plus DP was implemented in all intervention villages and the buffer zones; control villages received standard malaria interventions according to the Gambian National Malaria Control Program plans.

**Results:**

The MDA campaigns were carried out from August to October 2018 for the first year and from July to September 2019 for the second year. Statistical analysis will commence once the database is completed, cleaned, and locked.

**Conclusions:**

This is the first cluster randomized clinical trial of MDA with IVM plus DP. The results will provide evidence on the impact of MDA with IVM plus DP on malaria transmission.

**Trial Registration:**

ClinicalTrials.gov NCT03576313; https://clinicaltrials.gov/ct2/show/NCT03576313

**International Registered Report Identifier (IRRID):**

DERR1-10.2196/20904

## Introduction

Between 2000 and 2015, the burden of malaria decreased substantially in sub-Saharan Africa following the scale-up of insecticide-treated bed nets (ITNs), indoor residual spraying (IRS), and artemisinin-based combination therapy [1,2]. In The Gambia, between 2003 and 2007, the proportion of blood slides positive for malaria decreased by 74%, and hospital admissions for malaria by 81% [3,4]. Nevertheless, despite the high coverage of control interventions, malaria transmission has become increasingly heterogeneous [5-7]. Innovative interventions and tools to further reduce transmission and eliminate malaria are needed.

Mass drug administration (MDA), a full antimalarial treatment to all inhabitants of target communities regardless of their infection status, has been identified as a potential tool to further reduce transmission where coverage of vector control interventions is already high [8,9]. MDA with artemisinin-based combination therapy (ACT) reduces transmission by clearing asexual infections and early-stage gametocytes in asymptomatic, infected individuals [8,9]. Dihydroartemisinin-piperaquine (DP), thanks to its simple dosing schedule, long posttreatment prophylaxis period, good safety profile [10,11], and the fact that it is not used as a first-line antimalarial treatment in The Gambia, is a promising candidate for MDA. However, the effect of MDA with ACT on malaria is limited over time [12,13]. This limited impact is largely attributed to incomplete coverage and the persistence of malaria parasites in the mosquito population, with a smaller contribution to residual transmission of gametocytes remaining after ACT administration [14].

Combining an antimalarial treatment (DP) with an endectocide treatment able to reduce vector survival, ivermectin (IVM), could increase the impact of MDA and offer a new tool to reduce malaria transmission [15,16]. IVM is a broad-spectrum antiparasitic endectocidal drug, active against a wide range of parasites, including ectoparasites [17]. It reduces the lifespan of mosquitoes that feed on treated individuals [16], with conflicting data on a possible effect on parasite development in surviving mosquitoes [18-20]. A major advantage of IVM is that, since malaria vectors feed on more than one person, the effective coverage may exceed MDA coverage, especially if its mosquitocidal effect can be extended by repeated treatments. Repeated IVM dosing increases the duration of time that IVM concentration remains above the lethal concentration that kills 50% of mosquitoes [17]. A 3-day regimen of IVM 300 µg/kg is safe and has a mosquitocidal effect for *Anopheles gambiae s.s*. lasting approximately 28 days post treatment [16].

The community administration of IVM could reduce malaria transmission [19,21], providing a synergistic effect compared to MDA with ACTs alone [15]. Furthermore, IVM can reduce the prevalence of other parasitic infections, including ectoparasites and soil-transmitted helminths, which could be an important additional benefit and improve the cost-effectiveness of this intervention.

The mass drug administration of ivermectin and dihydroartemisinin-piperaquine as an additional intervention for malaria elimination (MASSIV) study is a cluster randomized trial that aims to evaluate the impact of MDA with IVM plus DP on malaria transmission in an area with high coverage of malaria control interventions, eg, ITNs, IRS, and seasonal chemoprevention (SMC). The trial was implemented in Basse, Upper River Region (URR) in The Gambia.

### Study Objectives

#### Clinical

The clinical objective is to determine whether three monthly rounds of MDA with IVM plus DP implemented over two malaria transmission seasons will reduce malaria transmission in communities with high coverage of malaria control interventions.

#### Entomological

The entomological aim is to determine whether three monthly rounds of MDA with IVM plus DP implemented over two malaria transmission seasons will reduce vector parity, a proxy for vector survival, in communities with high coverage of malaria control interventions.

#### Social Science and Health Economics

The social science and health economics aims are to (1) identify the most socially acceptable and sustainable way of achieving and maintaining high coverage of MDA with IVM plus DP, and (2) determine the costs and cost-effectiveness of this intervention compared to standard malaria control measures.

## Methods

### Study Design

The study is a cluster randomized trial (ClinicalTrials.gov, NCT03576313) that included a total of 32 villages (16 control and 16 intervention), located at least 3 km from each other. A buffer zone of ~2 km was created around all intervention clusters in a modified fried-egg design [22]. No buffer zone was created around control villages. MDA with IVM plus DP was implemented in all intervention villages and the buffer zones; control villages received standard malaria interventions according to the Gambian National Malaria Control Program’s (NMCP) plans.

### Study Setting

The study was conducted in the eastern part of The Gambia in the Upper River Region (URR). The region is open Sudanese savanna and covers an area of 1995 km^2^ [23]. Most of the residents are subsistence farmers. The climate is characterized by a long dry season from mid-October to mid-June, followed by a single short rainy season. Malaria transmission is highly seasonal, with most malaria cases occurring during the rainy season and immediately afterward, until December-January [23,24]. Malaria transmission has decreased substantially in The Gambia; however, it is higher in URR than in other regions [24]. The URR is also characterized by low vector density and high vector parity rate, indicating high vector survival, suggesting the existence of populations (or subpopulations) of mosquitoes able to escape standard vector control interventions [24].

### Selection of Villages and Informed Consent

A cross-sectional survey was carried out in November 2017, at the peak of malaria transmission, in 47 medium-sized (200-600 inhabitants) villages to select study villages with a baseline malaria prevalence of at least 10%. Following the survey, 32 villages with malaria prevalence determined by qPCR of at least 10% were included in the trial and randomly assigned to either the intervention or control arm using STATA, version 15; randomization was constrained such that the mean baseline prevalence in the intervention clusters was within ±10% of the prevalence in the control clusters.

After explaining the study objective and methods of the trial to the local authorities and the populations of the study villages through sensitization meetings in the local language, a census of the study population was carried out in November 2017. This was followed by individual consent procedures to obtain written informed consent for all willing residents in the study villages. Consent for children was provided by their parents/guardians; assent was sought for adolescents 12-17 years old.

Consent and enrolment procedures were carried out throughout the trial to obtain written informed consent from new residents and individuals missed previously by the research team due to absence at the time of the initial consenting and enrolment procedures.

A list of all residents in the study villages, by compound, including consent status, were generated and made available to the study team.

### Eligibility Criteria

The target population for MDA was all eligible individual residents in the intervention villages. During each MDA round, residents were (re)assessed for eligibility to receive IVM and DP. Participants had to meet all inclusion criteria and none of the exclusion criteria. Inclusion criteria were: (1) age/anthropometry, for IVM: weight ≥15 kg; for DP: age >6 months, (2) willingness to comply with trial procedures, and (3) individual written informed consent. The exclusion criteria for both IVM and DP were known chronic illnesses such as HIV, tuberculosis, hepatitis, and severe malnutrition. Additionally, for IVM only, exclusion criteria were (1) pregnancy (any trimester) or breastfeeding, (2) hypersensitivity to IVM, and (3) travel to *Loa loa* endemic countries (Central Africa); for DP, these were: (1) first-trimester pregnancy, (2) hypersensitivity to DP, and (3) taking drugs that influence cardiac function or prolong QTc interval.

### Trial Medication and Intervention

DP (Guilin Pharmaceuticals, China) was available as tablets of 320/40 mg and 160/20 mg piperaquine/dihydroartemisinin per tablet. DP was administered orally, once daily for three days, according to body weight per manufacturer’s guidelines. For participants unable to swallow the tablets, such as infants and young children, DP was crushed and mixed with water. The mixture was used immediately after preparation. If a participant vomited within 30 minutes of taking DP, the full dose was readministered; if a patient vomited within 30-60 minutes, half the dose was readministered. IVM (Laboratorio Elea SACIF y A, Argentina) was available as 6 mg tablets. It was given at 300-400 μg/kg/day once daily for three days (15.0-25.9 kg one tablet, 26.0-40.9 kg two tablets, 41.0-60.9 kg 3 tablets, 61.0-80.9 kg four tablets, 81.0-95.9 kg five tablets, ≥96 kg 6 tablets). Therefore, the total dose of IVM for each round was 900-1200 μg/kg. Before treatment, women of reproductive age (15-49 years) were asked to provide a urine sample to test for pregnancy. All treatments were supervised.

MDA was implemented each year and for two consecutive transmission seasons as three-monthly rounds starting from July (end of the dry season) to August and September (rainy season). The choice of implementing the intervention for two consecutive years was taken to estimate its cumulative effect and to monitor community participation over repeated intervention rounds.

Standard malaria control interventions were implemented in both intervention and control villages. These consisted of ITNs, IRS with pirimiphos-methyl (Actellic 300CS) done in mid-July 2019, just before the first MDA round of IVM and DP, prompt diagnosis and treatment with artemether-lumefantrine, SMC, and intermittent preventive treatment during pregnancy (IPTp). During the three rounds of MDA, SMC was not administered to children in the intervention villages eligible for MDA with IVM plus DP to avoid double antimalarial treatment. In these villages and during the MDA period, only children aged 3-6 months received SMC as they were not eligible for DP treatment. Nevertheless, all children aged 3-59 months received SMC one month after completing the last MDA round in intervention villages. The implementation of these malaria control interventions was done by the Regional Health Team and documented at the level of individual participants. Eligible children in control villages received SMC as planned by the NMCP.

### Outcome Measures

#### Clinical

The primary clinical endpoint was the prevalence of malaria infection measured by qPCR in all age groups [25] via a yearly cross-sectional survey at the peak of the transmission season in November.

Secondary endpoints included (1) incidence of clinical malaria estimated by passive case detection by recording all clinical cases from study villages (both intervention and control) attending the local health facilities or the community health worker when present; (2) prevalence of drug-resistant markers such as Pfcrt and Pfmdr-1 mutations [26], in malaria-positive blood samples collected during the annual cross-sectional survey; (3) serological markers of recent malaria infection and recent Anopheles exposure, both determined by detecting relevant antibody responses in blood samples collected during the annual cross-sectional survey; (4) intervention coverage as the proportion of eligible individuals having taken the investigational products.

Tertiary endpoints were the prevalence of bedbugs, headlice, scabies, and soil-transmitted helminths. Surveys were done yearly, before and after the MDA campaign.

#### Entomological

The primary entomological endpoint was the parous rate of *An. gambiae* *s.l*. females in each study group, determined on extracted ovaries [27]. The secondary endpoint was the duration of the IVM mosquitocidal effect, determined by the mortality of insectary colonized *An. coluzzii* after feeding on IVM-treated individuals on days 7, 14, and 21 post treatment. *An. coluzzii* mortality was recorded over 14 days after feeding.

#### Social Sciences and Economics

The economics and social sciences endpoints included: (1) MDA participation and acceptability, and (2) MDA costs and cost-effectiveness. For participation and acceptability, quantitative and qualitative research methods were used. For the former, in each intervention village, a cross-sectional survey was conducted between MDA years 1 and 2. For qualitative methods, in-depth interviews and focus group discussions with MDA participants, MDA decliners, village leaders, MDA field staff, and others were carried out throughout all trial activities. Information on the costs of malaria episodes to households and their management and the intervention costs was collected to estimate costs and cost-effectiveness. From these, the incremental cost-effectiveness ratio of the intervention versus control was calculated as costs per malaria-related DALY averted. Moreover, policy considerations around translating and scaling up the intervention were explored.

### Statistical Considerations

The sample size was based on two primary endpoints: malaria prevalence determined by qPCR at the peak of the malaria transmission season and the vector population’s parous rate. For malaria prevalence, we assumed the mean malaria prevalence in the control arm at peak transmission would be 15%; this is a conservative assumption, as, in November 2013, the prevalence in villages in eastern Gambia varied between 21.3% and 44.3% [24]. Based on an initial prevalence of 15% and assuming 95%, 86%, and 63% of eligible individuals receive 1, 2, or 3 courses of DP+IVM each year, prevalence would decrease by >90% assuming “ideal conditions” such as the absence of population movement, perfect adherence and no cluster spillover effects (human/vector movement from control to intervention clusters). However, such factors may influence the effect size of the intervention. Therefore, we used a more conservative effect size of 50% or prevalence from 15% to 7.5%. Assuming a conservative coefficient of variation of 0.5, 16 clusters per arm, 200 individuals per cluster recruited in the yearly cross-sectional survey, would have 90% power to detect a significant difference between study arms.

For the vector parous rate, the assumption was that the parous rate of *An. gambiae s.l.* would be 85% because, in 2013, it was 90.7% and 81.1% in the North Bank and South Bank of the URR, respectively [24]. Therefore, assuming the intervention would decrease parity from 85% to 75%, and a coefficient of variation of 0.25, dissecting 50 *An. gambiae s.l* per village would have 90% power to find a significant difference between arms.

### Randomization and Blinding

The unit of randomization was the village. Randomization to the intervention or the control arm was done by computer-based randomization. To prevent imbalance for potential confounding factors, restricted randomization to balance the arms on factors such as baseline prevalence, ITN coverage, and distance from health facilities was used [22].

Considering the nature of the trial (cluster randomized), the primary endpoint (malaria prevalence), and the logistical and ethical (treating thousands of individuals with placebo) challenges, we chose not to blind participating individuals. All laboratory staff involved in processing samples and evaluations contributing to any clinical or entomological endpoints were blinded to the allocation arm.

### Study Procedures and Evaluations

#### Clinical Evaluation

Passive case detection was established at local health facilities and the village level if community health workers were present. All suspected malaria cases, such as patients with fever or history of fever in the last 24 h without any other apparent illness, were investigated with an RDT (SD BIOLINE Malaria Ag Pf Standard Diagnostics); if positive, the patient was treated with artemether-lumefantrine, the first-line treatment in The Gambia. Axillary body temperature was measured using an electronic thermometer; a blood slide and blood spots were collected for later molecular analysis. Randomly selected individuals, regardless of temperature or history of symptoms, were included in the cross-sectional surveys to collect blood samples by finger prick and administer a short questionnaire on demographic characteristics, area of residence, recent travel history, and use of malaria control interventions and recent history of clinical malaria.

A cross-sectional survey to estimate the prevalence of ectoparasites, scabies, and helminths was carried out. In each cluster, 30 children aged 4-13 years, the age group at highest risk of these infections, were selected. Bedbugs were detected by visual inspection of the child’s bed using torchlight, and headlice were detected by visual search of the scalp. Scabies was identified by a complete physical examination of the body. A stool sample was taken to identify the presence of helminths, including *Strongyloides stercoralis, Trichuris trichiura*, *Necator americanus*, *Ancylostoma duodenale*, and *Ascaris lumbricoides* using PCR.

#### Entomological Evaluations

Both intensive and routine entomological surveys were performed. Intensive surveys were carried out 7-14 days after each MDA in all the intervention villages and for a similar period in 8 control villages randomly selected at the beginning of the study. The same control villages were sampled throughout the transmission season. Collections were carried out over three consecutive nights in six randomly selected houses. Monthly human landing catches (indoor and outdoor) were carried out in three houses for two nights (6 nights/month) to determine the landing rate, parity rate, sporozoite rate, and the entomological inoculation rate, and their potential reduction by IVM. Four collectors rotated between indoor and outdoor sites every 2 hours, from 7:00 pm to 7:00 am, in four selected villages per study arm. Routine surveys were carried out monthly, after the intensive surveys, between October and December, in all intervention and control villages using CDC light traps. [28]. In each village, six trapping nights/month with six indoor CDC light traps were positioned 1 m above the ground at the foot end of the bed (1 per house and protected by ITN) to obtain estimates of vector density, parity, and sporozoite rates. Parity was determined by dissecting 60 unfed *An. gambiae s.l*. (10 per trap per village) in droplets of phosphate-buffered saline (PBS) and read with a microscope at 10× field magnification. Sporozoite rate was determined by ELISA on the head and thorax of *An. gambiae s.l.*

To monitor the duration of the IVM mosquitocidal effect and potential host characteristics associated with lower efficacy, 40 randomly selected adults and 40 randomly selected children (2-10 years old) were recruited in one intervention and one control village near Basse field station, which has an insectary. Children were included in this component of the study because linear dosing based on total body weight may not achieve similar drug exposure in children as in adults, possibly a consequence of age-related changes in gastrointestinal motility, pH, or prehepatic expression of metabolic enzymes or transporters, resulting in increased absorptive capacity with age [29,30]. Only IVM-treated individuals were selected in intervention villages; all selected participants were asked to provide additional written informed consent. A 3-mL blood sample was collected at 7, 14, and 21 days post MDA. This blood sample was placed in a glass membrane feeding system and 2 cups (250 ml, covered at the top with netting), each containing 50 insectary-reared *An. gambiae s.s.* mosquitoes, were presented to the membrane feeder for 20 minutes [31]. The number of mosquitoes with an engorged abdomen was counted and those with unfilled/partially filled abdomens discarded. Mosquito survival was monitored daily until 14 days post feeding. To further understand heterogeneity in mosquito mortality, participants across different age groups, gender, and varying body mass index (BMI) were selected for these membrane feeding assays. Membrane feeding assays were performed using insectary-reared *An. coluzzii* at different age ranges.

#### Social Sciences Evaluations

A mixed-methods social science study was carried out prior to and throughout the trial. This study used ethnographic, qualitative, and quantitative methods to assess MDA coverage, potential bottlenecks to implementation, adherence, and acceptability among potential MDA participants. Before the initial MDA campaign, potential bottlenecks for the intervention were assessed, and recommendations were made to improve implementation in the first phase. These data helped fine-tune the intervention to local realities and engaged stakeholders to assure participation and long-term sustainability. In the second phase, exploratory research was conducted in all intervention and control villages to assess the MDA’s socio-cultural effects. Methods included observations of all trial components, in-depth and key-informant interviews, and focus group discussions. These methods were carried out in both the first and second years of the MDA. A quantitative household survey was conducted between years to assess findings from the first intervention year in more detail; these findings were then explored by additional qualitative and ethnographic work, including surveys, interviews, and focus group discussions in the second year.

#### Laboratory Evaluations

A finger prick blood sample was collected from all selected individuals; 40-50 µL (3 droplets) were blotted onto Whatman 3 MM filter paper and stored with a desiccant at 4°C. For the molecular detection of *P. falciparum* parasitemia, three 6-mm punches of dried blood spot, each representing approximately 8 μL blood, were added to a 96-deep well plate and used for DNA extraction. Extracted DNA was analyzed with varATS qPCR using 5 μL of extracted DNA per assay [24]. All qPCR output was analyzed using the BioRad CFX Manager software. Each plate contains two rows of *P. falciparum* standard dilution, used to make the quantification curve and allow quality control. For the serological analysis, serum was eluted from one 6-mm punch (~4 µL serum) from the filter paper. This punch was eluted in 200 µL PBS/azide to have one 1:50 predilution from which 10 µL were used on the Luminex plate. As controls for the immunoassay, BSA-coupled beads, a pool of negative sera (malaria naïve European sera), and positive sera (highly exposed Gambian sera from previous surveys) were added to each plate. Plates were analyzed and read using the MAGPIX [32]. Results were presented as the median fluorescence intensity.

### Data Capture, Management, and Analysis

Data for each participant were captured on electronic case report forms. Informed consent, demography, medical history, physical examination, vital signs, drug administration, adherence to study drugs, adverse events including serious adverse events, and laboratory evaluations were recorded.

All trial data were stored and managed within the REDCap trial database system, an application specifically designed to collect and store clinical trial data and customized for electronic data capture at the trial site. Analytical results for samples from the prevalence surveys were linked to the main trial database. When inconsistencies were found, these were checked against the original forms and subsequently amended in the dataset. All staff involved in the trial was trained on their specific tasks.

### Statistical Analysis

The primary clinical endpoint, malaria prevalence at the end of the second transmission season, will be compared between arms using random**-**effects logistic regression, and a random effect for study village will be included to account for clustering. An odds ratio, 95% CI, and *P* value will be presented. Malaria prevalence at the end of the first transmission season will be analyzed separately and in the same way.

The primary entomological endpoint, vector parity, will be compared between arms using random-effects logistic regression, and a random effect for study village will be included to account for clustering. A fixed effect for study month and year will be included. An odds ratio, 95% CI, and *P* value will be presented. Incidence will be compared between arms using random-effects Poisson regression, and a random effect for study village will be included to account for clustering. An offset for village population and a fixed effect for the study year will be included. A rate ratio, 95% CI, and *P* value will be presented. Mosquito mortality, a secondary entomological endpoint, will be determined by Kaplan-Meier estimates. Kaplan-Meier plots of mosquito survival will be presented for each age group and timepoint. A comparison between the arms will be made using survival analysis with an exponential survival model. A random effect (shared frailty) will be included for each human participant to account for correlated outcomes on mosquitoes fed on the same participant.

Other entomological endpoints: (1) Mosquito density will be compared between arms using random**-**effects negative binomial regression, and a random effect for study village will be included to account for clustering. A fixed effect for study month and year will be included. An odds ratio, 95% CI, and *P* value will be presented; (2) Sporozoite rates in field-caught mosquitoes will be compared between arms using random**-**effects logistic regression, and a random effect for study village will be included to account for clustering. A fixed effect for study month and year will be included. An odds ratio, 95% CI, and *P* value will be presented.

### Quality Management

An independent data and safety monitoring board (DSMB) was appointed to advise the sponsor and investigators on the trial’s safety issues. Study physicians and nurses were trained to monitor and report all adverse events, including serious adverse events associated with treatment. Before each dose, adverse events that occurred were systematically documented by medical terms, start and end date, severity and seriousness, relation to the study drug, and outcome. The relationship to the study drugs was determined based on temporal association and clinical judgment. The study nurses visited each household in intervention villages 7 days after starting treatment to monitor and document any adverse events in the study population. Serious adverse events were reported to the DSMB, the local ethics committee, and the London School of Hygiene and Tropical Medicine (LSHTM) Ethics Committee. Analysis of adverse events was restricted to the intervention arm because the study population in the control arm was not treated with the investigational products.

### Ethical Approval

The protocol, informed consent documents, and patient information sheets have been reviewed and approved by The Gambia Government/MRC Joint Ethics Committee (ref number: 1593) and the London School of Hygiene and Tropical Medicine Ethics Committee (ref number: 15823).

## Results

The MDA campaigns were carried out from August to October 2018 for the first year and from July to September 2019 for the second year. Statistical analysis will commence once the database is completed, cleaned, and locked.

## Discussion

### Principal Findings

IVM is a promising tool to complement the current effort to interrupt malaria transmission. It targets malaria vectors regardless of biting behavior (it kills exophagic and exophilic mosquitoes, including early biting vectors) and reduces vectorial capacity [15,33]. Research has shown that IVM is a potentially valuable addition to the existing tools to decrease malaria transmission [15,16,18,33,34]. Modeling showed that three monthly rounds of MDA with IVM at 300 μg/kg/day daily for three days with 70% coverage would reduce clinical incidence by 70% and prevalence by 34% in a highly seasonal moderate transmission setting [15]. The magnitude of the impact is predicted to be higher for MDA of IVM plus DP, resulting in a reduction of clinical incidence by 75% and prevalence by 64% [15]. Nevertheless, this trial compares MDA of IVM plus DP to standard malaria control interventions and cannot distinguish the IVM effect from the DP effect. The proposed trial will evaluate the impact of MDA with IVM plus DP.

Cluster randomized trials are the gold standard design to assess the community-level effect of an intervention [22]. Key considerations for the design are cluster size, spatial separation, and movement of individuals between intervention and control clusters [35,36]. Individual movement could lead to contamination or spillover, which would affect the interpretation of the intervention’s efficacy [35]. We have defined a buffer zone around intervention clusters to reduce the spillover effect and ensure that intervention and control clusters are spatially well separated. The intervention (MDA with IVM plus DP) was carried out in intervention clusters and buffer zones to minimize contamination of the intervention villages. The entomological endpoint was one of the trial’s primary objectives, and buffer zones were created to minimize contamination of intervention villages by vectors from control villages.

Measuring and detecting changes in malaria transmission requires appropriate metrics to assess the community-wide impact of an intervention [37]. Several metrics measuring different facets of malaria transmission have been proposed, including entomological inoculation rate, slide positivity rate, parasite prevalence, disease incidence, sporozoite rate, vectorial capacity, and serological markers of mosquito and malaria exposure [37-39]. All these metrics have intrinsic limitations to their precision and accuracy [38]. Nevertheless, parasite prevalence is a suitable direct estimate of malaria infection in low transmission settings [37,38].

Parasite prevalence, the metric designed to measure the proportion of individuals found with parasites in their blood, varies by the methods used [37], including microscopy, RDT, and PCR [37,39]. Microscopy and RDTs may miss infections of low parasite density that represent a large proportion of infections in a low-transmission setting. Parasite prevalence at the peak of the transmission season and determined by PCR can provide an accurate estimate of the community’s parasite reservoir and the effect of the intervention [38,40]. This trial’s primary endpoint is parasite prevalence by PCR in all age groups through a community-based random sampling survey at the peak of the transmission season.

Successful MDA campaigns require high coverage and good compliance [41]. Understanding the local context and attitudes within the trial communities, including the communities’ acceptability of the intervention, is key to achieving the necessary coverage and compliance to meet the desired clinical and epidemiological results. For example, healthy individuals, some of whom may be malaria-infected but asymptomatic, may not be ready to accept medications for a disease they are currently not affected by, and this could negatively impact trial coverage. Engaging communities to establish the most effective, ethical, and sustainable ways to implement these community-targeted interventions is vital. Therefore, community acceptance was assessed using a mixed-method design. During and after each round of MDA, acceptability was evaluated, and the findings informed the implementation of subsequent rounds.

To our knowledge, this is the first cluster randomized trial with a 3-day regimen of 300 µg/kg IVM (high-dose regimen). Strengths of the study include its design as an adequately powered cluster randomized trial, the setting in an area with high coverage of ITNs, IRS, and SMC, and where there is prompt and effective treatment with ACTs. Another major strength is the provision of safety data for the high dose IVM administered with DP. The trial is limited by its inability to determine the impact of IVM alone as this treatment is administered with DP, and there is no DP-only arm.

### Conclusion

This study will be the first cluster randomized clinical trial of MDA with IVM plus DP. The results will provide evidence on the effect of MDA with IVM plus DP on malaria transmission.

## Abbreviations

ACT: artemisinin-based combination therapy
CDC: Centers for Disease Control and Prevention
DP: dihydroartemisinin-piperaquine
DSMB: data and safety monitoring board
IPTp: intermittent preventive treatment during pregnancy
IRS: indoor residual spraying
ITN: insecticide-treated net
IVM: ivermectin
LSHTM: London School of Hygiene and Tropical Medicine
MDA: mass drug administration
PBS: phosphate-buffered saline
PCR: polymerase chain reaction
qPCR: quantitative polymerase chain reaction
QTc: rate corrected QT interval on an electrocardiogram
RDT: rapid diagnostic test
SMC: seasonal chemoprevention
URR: upper river region
varATS: var Gene acidic terminal sequence
